# Primary care must be prioritised to achieve universal palliative care

**DOI:** 10.3399/BJGP.2025.0344

**Published:** 2025-10-01

**Authors:** Sarah Mitchell, Claire Fuller, Amanda Doyle, Lucy Ziegler

**Affiliations:** 1 NHS England, London, UK; 2 Academic Unit of Palliative Care, Leeds Institute of Health Sciences, School of Medicine, University of Leeds, Leeds, UK

## Universal palliative care can only be achieved through primary care

More people worldwide are living with serious illness and the suffering associated with it, meaning that the need for good palliative care and end-of-life care is rising.^1^ The progression of the Terminally Ill Adults Bill through parliament has sparked new media and political interest in palliative and end-of-life care. Narratives pervade about challenges and failures in the delivery of palliative and end-of-life care, and the associated pain and distress experienced by patients.

In England, 1% of the population dies every year, over 500 000 people, and this is expected to increase to over 640 000 by 2040.^2^ More people are dying in the community, with 57% of deaths occurring in community settings in 2023/2024, including in people’s homes (28%), care homes (22%), hospices and other residential settings (7%). Palliative care is an approach focused on improving quality of life for people with life-threatening illness, through the timely identification and management of physical, psychological, social, and spiritual (or cultural) needs.

Primary care, community services, and hospices collectively receive less than 10% of the £9.5 billion of public expenditure on health care for people in the last 12 months of their life in England.^3^ Despite this, many GP and primary care colleagues are deeply committed to the provision of palliative care to their patients. The ability to deliver continuity of care in primary care is key. Continuity in primary care is associated with less frequent use of emergency hospital care towards the end-of-life for patients of all ages, suggesting improved patient experience as well as positive healthcare system outcomes.^4,5^ GPs and primary care teams know the needs of the community they serve and are uniquely placed to provide holistic and coordinated care in response to patient need, not diagnosis. Universal palliative care will not be achieved without them.^6^

This Editorial responds to changes and concerns raised in relation to palliative care ‘registers’ in the 2025/2026 GP Contract, in which all income-protected Quality and Outcomes Framework (QOF) registers were retired.^7^ This change has caused concern among the palliative care sector at a national level, with the recently published Parliamentary Commission report *Palliative Care and End-of-Life Care: Opportunities for England*, stating that this change will *‘run the risk of reducing pressure on GPs to identify patients with palliative care needs’*.^8^

## Moving on from the idea of a palliative care ‘register’

The Palliative Care Register has been a feature of the QOF since 2008, worth three QOF points (or just over £600 per practice per year). The quality of GP palliative care registers receives little scrutiny and the impact on patient care (or not) is largely unknown. Much more research is needed to understand the provision of palliative care in primary care. The existence of palliative care registers has not addressed longstanding inequity of access to specialist palliative care services. Patients with cancer, from more affluent areas, and who are White British, are more likely to receive specialist palliative care services than those with non-malignant disease, from more socioeconomically deprived areas, and from minority ethnic backgrounds.^9^ To date there has not been any work to understand whether this inequity is specific to specialist palliative care services, or whether this is also reflected in the identification of palliative care need and provision of care in general practice.

## Opportunity for positive change

Despite current pressures, general practice is already responding to the rising patient and population need for palliative and end-of-life care. There was a 27% rise in rates of identification of palliative care need in primary care in England in 2023/2024 compared with 2022/2023, from 0.46% of the population to 0.55%.^10^ Our understanding at NHS England is that this is due to the drive and commitment of clinical leaders, in GP practices, integrated care systems, and across regions. Clinical leaders have led a variety of innovations and initiatives to improve the identification of palliative care needs and associated coding in patient records, alongside education and training to improve understanding of palliative care. In general practice, this increase in identification has occurred despite time and resource pressures, and alongside the delivery of more appointments than ever.

It is estimated that between 0.7% and 0.9% of the population (350 000–450 000 people) could benefit from palliative care each year.^2^ Rates of identification of palliative care need across Integrated Care Systems vary. In some places, rates are as high as 0.89%, while in others the rate is much lower, around 0.22%.^10^ There is opportunity to share learning and adapt initiatives by working together through regional and national networks. Changes in the GP contract provide opportunity to push forward with more positive change. Most importantly, there has been an uplift in the global sum (per patient payment to general practice), the biggest increase in investment into general practice in over 10 years. A new domain in the Capacity and Access Improvement (CAIP) payment is intended to incentivise primary care networks (PCNs) to take a population health approach and risk stratify their patients, to identify those who would benefit most from continuity of care.^7^ This requires including patients with palliative care needs and those approaching the end of life, and aligning with guidelines for neighbourhood health, which prioritise groups of patients who could most benefit from continuity and specifically reference patients with palliative care and end-of-life care needs.^11^

## Putting patients first

Identification of patients’ palliative care needs and recognition that a patient is approaching the end of life is only meaningful if it positively impacts their care. This is a time when care and compassion matter most, but experiences of identification are highly variable from a patient perspective.^12^ The core elements of good palliative care and high-quality end-of-life care must follow. Primary care clinicians have the expert generalist skills and potential to deliver most of this, including compassionate communication and care planning, prescribing and de-prescribing, planning for out-of-hours and referrals to other services including specialist palliative care, where necessary and available. These are complex tasks that take time, requiring regular review, compassion, communication skills, and effective management of clinical risk and uncertainty. Continuity of care is essential.

## Moving forward

Prioritising the role of primary care in palliative and end-of-life care in practice, policy, and research is critical to improving patient care, achieving the vision set out in the government’s new 10 Year Health Plan and the delivery of neighbourhood health.^13^ General practice and primary care should build on good practice and continue to adapt to meet rising patient and population need, using all available contractual levers to support this, with research and investment in rigorous evaluation of change to inform future policy.
